# Paraganglioma of the Spermatic Cord: A Case Report and Literature Review of an Uncommon Entity

**DOI:** 10.7759/cureus.111628

**Published:** 2026-06-27

**Authors:** Jaydeep N Pol, Priya Hombalkar, Anand Bhosale, Ashna Agarwal, Nitin Hombalkar

**Affiliations:** 1 Pathology, Mahatma Gandhi Cancer Hospital, Miraj, IND; 2 General Surgery, Government Medical College, Miraj, IND; 3 Pathology, DY Patil University School of Medicine and Pushpalata DY Patil Hospital, Pune, IND; 4 General Surgery, Rajarshee Chhatrapati Shahu Maharaj Government Medical College, Kolhapur, IND

**Keywords:** metastasis, neuroendocrine tumor, paraganglioma, pheochromocytoma, spermatic cord

## Abstract

Paragangliomas are rare neuroendocrine tumors that arise from extra-adrenal chromaffin cells. While their occurrence in the urogenital tract is itself uncommon, involvement of the spermatic cord is exceptionally rare. Because of their nonspecific clinical presentation, these tumors are often misdiagnosed as more common inguinoscrotal lesions. Herein, we present the case of a 13-year-old male patient who presented with a gradually enlarging, non-tender inguinal mass. The lesion was surgically excised, and histopathological examination revealed a well-circumscribed tumor composed of polygonal cells arranged in the characteristic Zellballen pattern, showing minimal nuclear atypia and low mitotic activity. Immunohistochemistry confirmed the diagnosis, with the tumor demonstrating diffuse positivity for synaptophysin and chromogranin A. The postoperative course was uneventful, and the patient remains disease-free at three years of follow-up. This case is particularly noteworthy given its occurrence in the pediatric age group and adds to the very few reported cases of spermatic cord paraganglioma in the literature. It highlights the importance of including this rare entity in the differential diagnosis of inguinoscrotal masses and reinforces the need for histopathological and immunohistochemical evaluation for definitive diagnosis. Given the potential for malignant behavior, complete surgical excision with long-term surveillance remains essential.

## Introduction

Paragangliomas are neuroendocrine tumors arising from chromaffin cells in the sympathetic and parasympathetic paraganglia [1]. They are most commonly found in the head and neck regions, in sites like the carotid body, jugulotympanic paraganglia, vagal nerve, ganglion nodosum, and laryngeal paraganglia. They are also found along the cranial and thoracic branches of the glossopharyngeal and vagus nerves. They can be seen at uncommon sites like para-aortic, peri-adrenal, inter-aortocaval, and para-canal retroperitoneal sites and organs, namely, the lung, heart, mediastinum, thyroid, parathyroid, pituitary, pancreas, and liver [2,3]. They can arise where the paraganglia reside in the adult tissues or during embryogenesis. The probability of metastatic diseases can be considered when there is a known primary focus in a patient with no germline mutation to multifocal disease. The five-year survival rate for metastatic disease was found to be 63% in a large meta-analysis. Primary paraganglioma of the spermatic cord is very rare and was first reported by Eusebi and Massarelli in 1971 [4]. Paragangliomas throughout the body have a typical arrangement of chief cells into well-defined cell nests, often referred to as Zellballen, surrounded by a thin layer of Sertoli cells [5]. About 98% of overall paragangliomas are found in the abdomen, and 85-90% occur in the adrenal glands [6]. Paragangliomas may turn out to be malignant 40-50% of the time, as opposed to pheochromocytomas, at 10% [6]. The patient did not have any associated systemic disorder. Around 30% of these tumors may arise as part of hereditary syndromes, the most common being Carney triad, von Hippel-Lindau (VHL) syndrome, multiple endocrine neoplasia type 2 (MEN2), and neurofibromatosis type 1 (NF1) [7].

## Case presentation

A 13-year-old male patient presented to the surgical outpatient department of a tertiary care center with a painless left scrotal swelling that had been progressively enlarging over a period of three weeks. There was no antecedent history of trauma, and no swelling was noted at any other site. Family history was remarkable for an extra-adrenal paraganglioma in his younger sibling, who had undergone surgical excision one year prior. On physical examination, a firm, non-tender mass was palpable along the left spermatic cord. Contrast-enhanced computed tomography revealed a well-defined mass overlying the left spermatic cord. Routine hematological and biochemical investigations were within normal limits, and serum testicular tumor markers, including alpha-fetoprotein, human chorionic gonadotropin, and lactate dehydrogenase, were unremarkable.

The patient was planned for surgical excision of the mass. Intraoperatively, manipulation of the tumor precipitated a hypertensive crisis, with blood pressure rising acutely to approximately 220/140 mmHg. A sublingual antihypertensive agent was promptly administered, and rapid control of the tumor pedicle was achieved. Blood pressure normalized immediately following pedicle ligation. Upon complete excision of the mass, a transient hypotensive episode was noted (90/60 mmHg), which resolved with conservative management. The left testis was identified intraoperatively as being distinctly separate from the tumor mass. The postoperative course was uneventful, and the patient recovered without complications.

**Figure 1:**
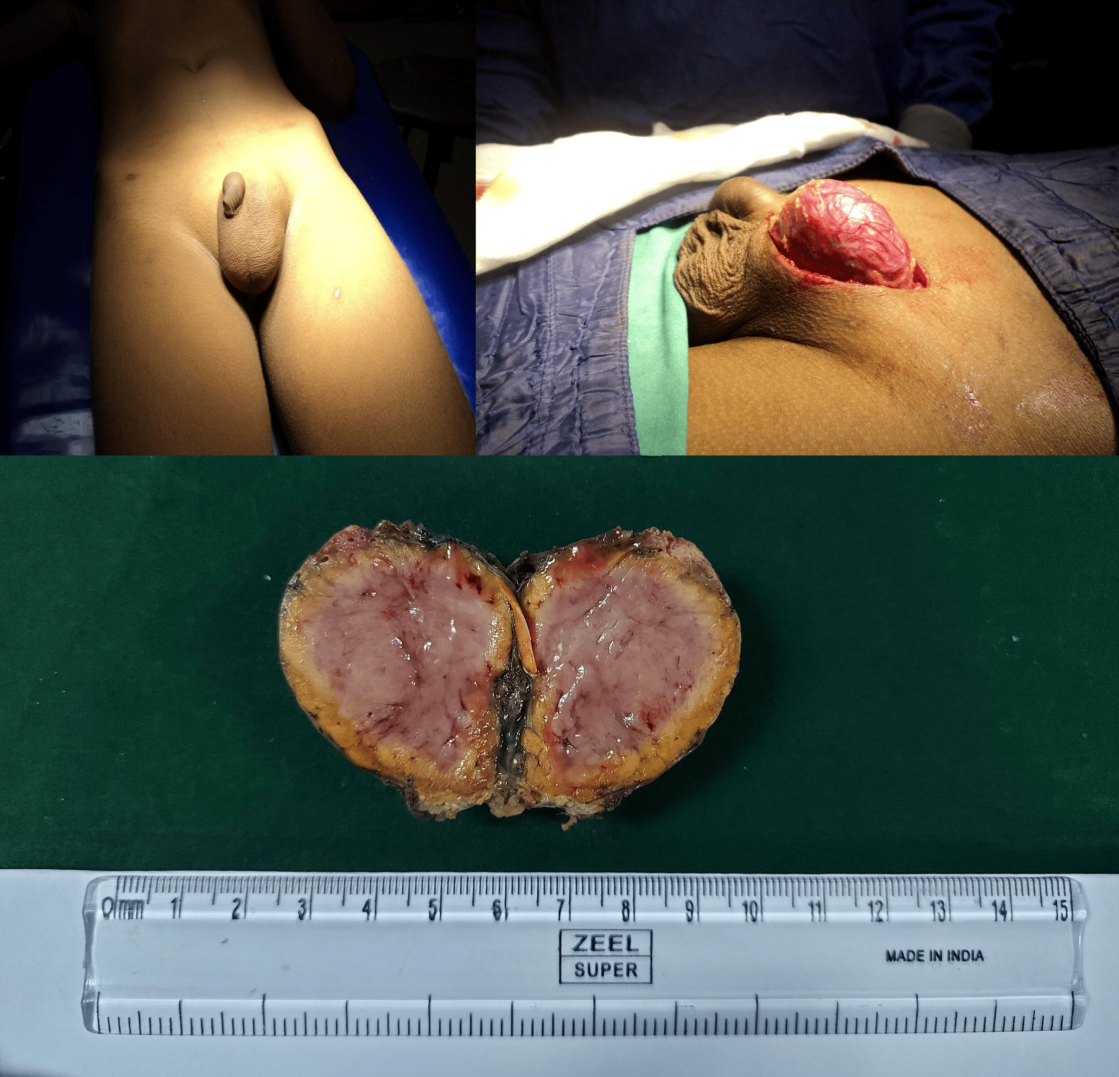
(A) Clinical image of the left spermatic cord swelling in the inguinal region. (B) Intraoperative image showing the left spermatic cord mass separate from the left testis. (C) Gross image of the resected mass showing an encapsulated solid tumor with a gray-tan appearance.

On gross examination, the resected left spermatic cord mass measured 4×3×2.9 cm. The external surface was rough with the intact capsule. On serial sectioning, an encapsulated grayish-tan solid tumor was noted (Figure 1).

**Figure 2:**
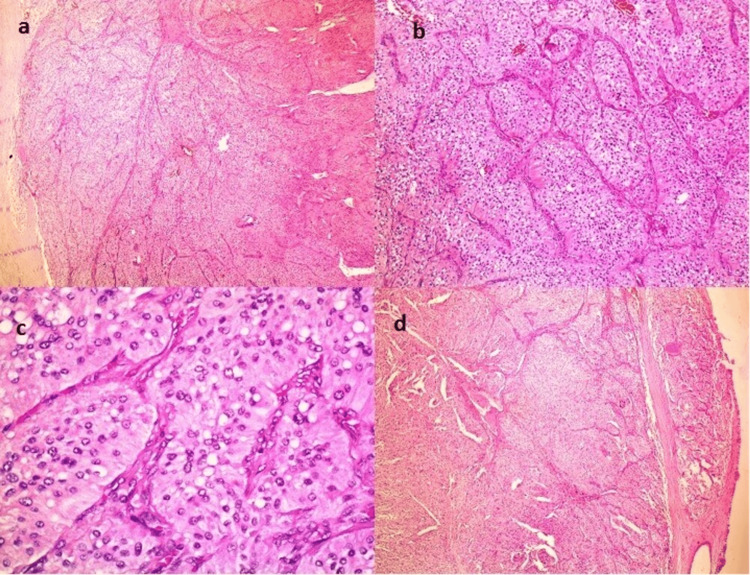
H&E images of the tumor showing (a) an encapsulated tumor, (b) the tumor cells arranged in nests creating a typical Zellballen pattern, (c) the tumor cells with minimal nuclear atypia, and (d) focal capsular invasion. H&E: (a) and (d) ×40, (b) ×100, and (c) ×400. H&E: hematoxylin and eosin

The microscopy revealed a partially encapsulated tumor composed of round to polygonal cells with round nuclei and eosinophilic or clear cytoplasm arranged in nests and clusters and in a typical Zellballen pattern. Nuclear atypia was minimal. No tumor necrosis, areas of hemorrhage, or spindle cell elements were noticed. No increased cellularity or composite tumor elements were seen. Mitotic count was <2 mitoses/10 high-power field (HPF). Focal capsular invasion was noted; however, perineural and lymphovascular invasions were not seen (Figure 2).

**Figure 3:**
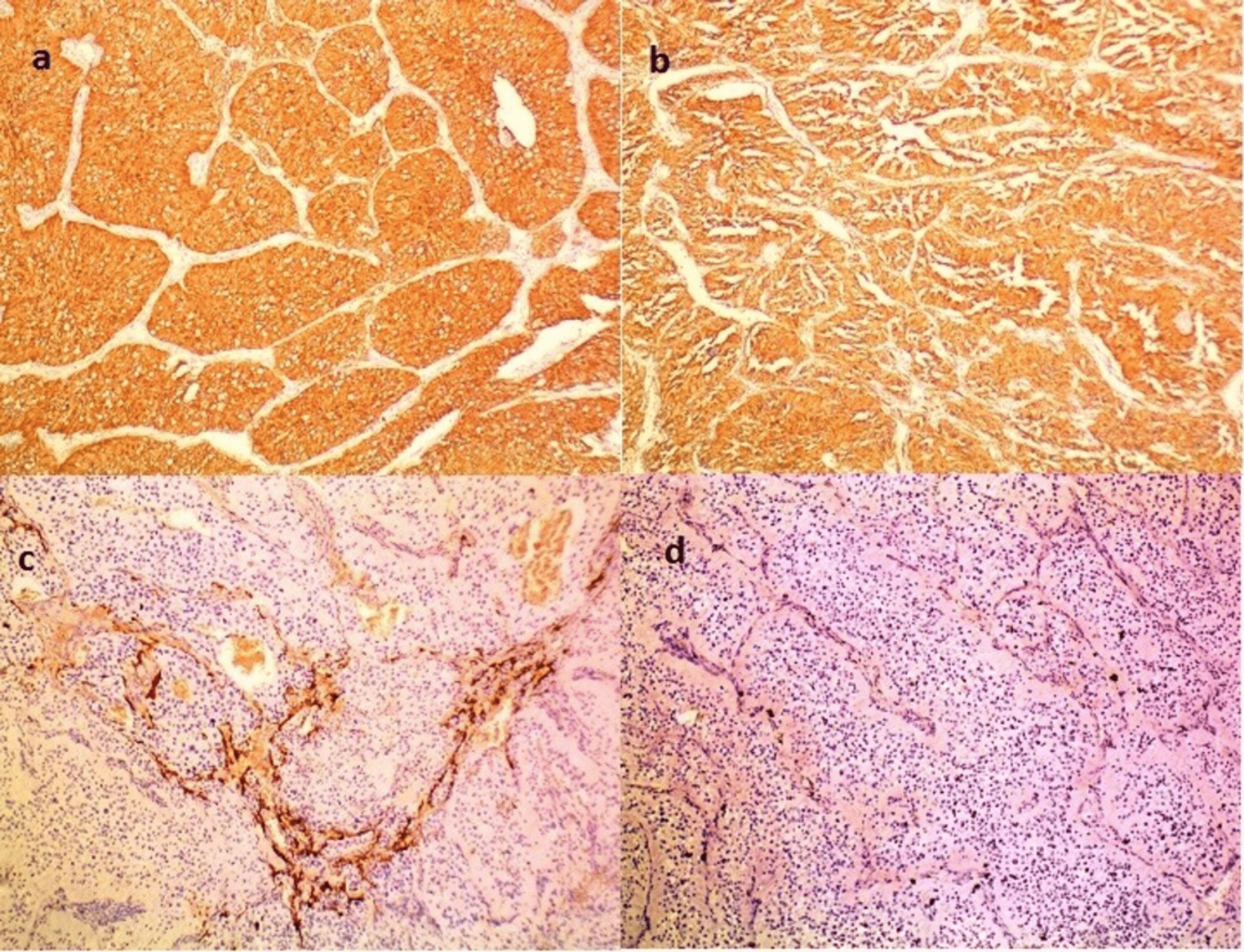
IHC images showing the tumor cells with bright and diffuse expression of (a) synaptophysin, (b) chromogranin A, and (c) reticulin stain accentuating the nested pattern of tumor cells and (d) the tumor cells negative for CK. IHC: (a-d) ×100. IHC: immunohistochemistry; CK: cytokeratin

Tumor cells were not present at the resection margin. Histopathological examination suggested the possibility of a paraganglioma of the left spermatic cord. On immunohistochemistry, the tumor cells showed diffuse and strong expression of synaptophysin and chromogranin A. Reticulin stain was seen around the nests of tumor cells highlighting the Zellballen pattern (Figure 3). The immunohistochemistry confirmed the diagnosis of paraganglioma of the left spermatic cord.

The patient was discharged after seven days of uneventful hospital stay and postoperative care. He is asymptomatic and disease-free three years after the resection of the tumor.

## Discussion

Paragangliomas are non-epithelial neuroendocrine neoplasms that arise from the paraganglia of the sympathetic and parasympathetic nervous systems [2]. If they are seen in the adrenal medulla, they are known as pheochromocytomas, and if discovered at any other locations, they are called sympathetic paragangliomas [1].

During embryogenesis, these cells are seen to arise from neural crest cells, which later on become chromaffin cells. They migrate along the sympathetic ganglia in the paravertebral axis and settle in the adrenal medulla. The second most common location is the organ of Zuckerkandl at the bifurcation of the aorta. They are also found in various other sites such as the urinary bladder [2]. The incidence rate of paraganglioma in the urogenital system is very low, especially in the spermatic cord [6].

The radiological and histopathological investigations of the paragangliomas of the spermatic cord overlap with the features of some other tumors. Ultrasound findings are similar to those of solitary fibrous tumors and schwannomas. The histopathological picture may mimic carcinoid tumors, Sertoli cell tumors, or metastatic paragangliomas [5]. Paraganglioma of the spermatic cord typically demonstrates a characteristic Zellballen architecture composed of well-circumscribed nests of chief cells separated by a delicate fibrovascular stroma. The cells possess finely granular eosinophilic cytoplasm and round nuclei with stippled "salt-and-pepper" chromatin, features that may overlap with neuroendocrine neoplasms. In contrast, granular cell tumors are composed of diffuse sheets or ill-defined clusters of large polygonal cells with abundant coarse granular eosinophilic cytoplasm, indistinct cell borders, and relatively bland nuclei, lacking the prominent vascular nesting pattern seen in paraganglioma. Similarly, carcinoid tumors exhibit trabecular or insular arrangements with uniform cells showing neuroendocrine chromatin, while Sertoli cell tumors more commonly display tubular, cord-like, or trabecular architecture within a fibrous stroma and lack the abundant granular cytoplasm characteristic of granular cell tumors. Immunohistochemistry is particularly useful in resolving these differentials. Paragangliomas and carcinoid tumors show positivity for neuroendocrine markers such as chromogranin A and synaptophysin, whereas paragangliomas additionally demonstrate S100 positivity restricted to sustentacular cells surrounding the nests. Sertoli cell tumors are typically positive for inhibin, calretinin, and steroidogenic factor 1 (SF1). In comparison, granular cell tumors show strong diffuse S100 positivity within the tumor cells themselves and are generally negative for chromogranin A and synaptophysin. Therefore, careful assessment of morphology together with an appropriate immunohistochemical panel is essential for accurate diagnosis.

In the latest edition of the WHO classification for urological tumors in 2016, paraganglioma of the spermatic cord was established as ICD-O Code 8963/1, which is believed to be a borderline tumor with malignant potential. The tumor was staged as TNM stage pT1 per the American Joint Committee on Cancer (AJCC) TNM staging system for paragangliomas, 8th edition [5,8]. Patients with metastatic paraganglioma have a short survival period. But diagnosing a metastatic lesion of paraganglioma is difficult due to its spread in non-chromaffin tissues.

A literature review was conducted through PubMed and the National Center for Biotechnology Information database using the keyword search term paraganglioma and spermatic cord. All cases of paraganglioma of the spermatic cord published hitherto were included. Reports published in languages other than English and those without an English abstract were excluded. This yielded only 16 cases of paraganglioma of the spermatic cord, with the age of presentation ranging from 28 to 69 years and a mean age of 45 years and a size ranging from 1.5 cm to 10 cm and a mean of 3.2 cm [3,5,6,9]. This case is the first case found in the pediatric age group and the first case reported in India. The presence of metastasis could not be determined in one case, while one case showed evidence of distant metastasis.

To further contextualize the clinicopathological features of paraganglioma observed in the present case, a comparative analysis with previously reported cases is presented. Paragangliomas demonstrate considerable heterogeneity in terms of anatomical location, clinical presentation, histomorphology, and immunohistochemical profile, particularly when arising at rare extra-adrenal sites. A structured summary of the selected reported cases is provided in Table 1, highlighting key demographic, pathological, and diagnostic features to facilitate comparison with the current case.

**Table 1:** Comparative clinical features of spermatic cord paraganglioma cases NED: no evidence of disease; Syn: synaptophysin; CGA: chromogranin A; CK: cytokeratin; IHC: immunohistochemistry; AFP: alpha-fetoprotein; HCG: human chorionic gonadotropin; LDH: lactate dehydrogenase; SDHB: succinate dehydrogenase subunit; FDG PET: fluorodeoxyglucose positron emission tomography; BP: blood pressure; CT: computed tomography; HPF: high-power field

Feature	Garaffa et al. [3]	Gontarz et al. [6]	Sun et al. [5]	Present study
Age (years)	Adult (28-69 range)	Adult (28-69 range)	Middle-aged	13
Sex	Male	Male	Male	Male
Side	Right	Not specified	Right	Left
Duration of symptoms	Not specified	Not specified	2 years	3 weeks
Presenting complaint	Inguinal/paratesticular mass	Inguinal/scrotal mass	Scrotal mass	Progressive, non-tender scrotal swelling
Family history	Not reported	Not reported	Not reported	Sister with extra-adrenal paraganglioma
Tumor size (cm)	Within a 1.5-10 cm range	Within a 1.5-10 cm range	Within the reported range	4×3×2.9
Catecholamine secretion	Non-functional	Non-functional	Non-functional	Functional (BP spike 220/140 mmHg)
Imaging	FDG PET: avid lesion in the right spermatic cord	Ultrasound	Ultrasound: abundant blood supply, clear boundaries	CT: well-defined mass over the left spermatic cord
Tumor markers	Not specified	Not specified	Not specified	AFP, HCG, LDH (normal)
Histopathology	Zellballen pattern	Zellballen pattern	Paraganglioma morphology	Zellballen pattern; minimal atypia; <2 mitoses/10 HPF; focal capsular invasion
IHC markers	Syn (+), CGA (+)	Syn (+), CGA (+), S100 (+)	CGA (+), Syn (+), CD56 (+), SDHB (+), S100 (+)	Syn (+), CGA (+), CK (−), reticulin (+)
Treatment	Surgical excision	Surgical excision	Surgical excision	Complete surgical excision
TNM stage	Not specified	Not specified	Not specified	pT1
Follow-up outcome	NED	NED	NED	NED
Metastasis	None	None	None	None

Earlier investigators have put forth systems like the Pheochromocytoma of the Adrenal Gland Scaled Score (PASS) grading system and the Grading System for Adrenal Pheochromocytoma and Paraganglioma (GAPP), essentially consisting of analysis of the histopathological features to foresee the metastatic potential of paragangliomas [10]. Although it is easily diagnosed by routine histology combined with immunohistochemistry, the rapid intraoperative frozen diagnosis can be challenging. Gene detection is recommended only if necessary. Early diagnosis is helpful in the choice of operation mode and the prevention and control of intraoperative risk [5].

The medical therapies for inoperable cases of paraganglioma include ablative therapy, external beam radiotherapy, and radiosurgery for localized lesions. Radionucleotide therapies, chemotherapies, and molecular targeted therapies are being trialed for systemic therapies [11].

Diagnosing paragangliomas is important as they are borderline tumors with malignant potential. A few tumors may show metastasis at the time of presentation. Surgical excision is the first and foremost intervention necessary in these cases. Whether to use chemotherapy or radiotherapy is still a debate. But close follow-up is necessary in such cases [5].

## Conclusions

Paraganglioma should be considered as an important differential diagnosis in cases of inguinal or scrotal swellings presenting in unusual locations. Diagnosing paragangliomas is important as they are borderline tumors with malignant potential. The case was presented for its rarity, and since it is usually unthought-of at this unusual location, it can be overlooked and mistaken for its mimics.
